# Experimental Research on Measuring the Concentration of CO_2_ in Gas-Liquid Solution Based on PZT Piezoelectric-Photoacoustic Spectroscopy

**DOI:** 10.3390/s22030936

**Published:** 2022-01-25

**Authors:** Hongquan Zheng, Yunlong Li, Yang Chen, Zhentao Wang, Jingmin Dai

**Affiliations:** School of Instrumentation Science and Engineering, Harbin Institute of Technology, Harbin 150001, China; zhenghongquan@stu.hit.edu.cn (H.Z.); 20S001030@stu.hit.edu.cn (Y.L.); 16B901006@stu.hit.edu.cn (Y.C.); 20B901047@stu.hit.edu.cn (Z.W.)

**Keywords:** gas-liquid solution, direct measurement, photoacoustic spectroscopy, PZT piezoelectric effect

## Abstract

The feasibility of a scheme in which the concentration of CO_2_ in gas-liquid solution is directly measured based on PZT piezoelectric-photoacoustic spectroscopy was evaluated. The existing device used for the measurement of gas concentration in gas-liquid solution has several limitations, including prolonged duration, loss of gas, and high cost due to the degassing component. In this study, we developed a measuring device in order to solve the problems mentioned above. Using this device, how the intensity of the photoacoustic signal changes with the concentration of CO_2_ was demonstrated through experiment. The impact that variation of the laser modulation frequency has on the photoacoustic signal was also studied. Furthermore, the experimental data generated from measuring the concentration of CO_2_ in gas-liquid solution was verified for a wide range of concentrations. It was found that, not only can the error rate of the device be less than 7%, but the time of measurement can be within 60 s. To sum up, the scheme is highly feasible according to the experimental results, which makes measurement of the concentration of a gas in gas-liquid solution in the future more straightforward.

## 1. Introduction

At the present time, the technology of measuring trace gas concentration is of great importance in the fields of environmental monitoring, power systems, medical assessment and others [1,2,3]. In 2020, Dan C. Dumitras et al. described in detail the application of near-infrared photoacoustic spectroscopy in human breath analysis, which provided different sampling methods for human disease screening [4]. Photoacoustic spectroscopy is commonly applied in the measurement of trace gas concentrations due to its quick response, high reliability, and strong anti-interference ability [5,6,7]. In 2017, Klemens, M. et al. measured the concentration of N_2_O in a gas-liquid solution (wastewater) using photoacoustic spectroscopy in an experimental setup [8]. In 2020, Liu et al. measured the concentration of CO_2_ in gas-liquid solution (transformer oil) using FTIR-PAS technology, with the sensitivity of the sensor reaching 4 μL/L [9]. However, previous devices used for the measurement of gas concentration in gas-liquid solution based on photoacoustic spectroscopy need to be degassed during the process of measurement because the capacitive microphone which acts as the main acoustic measurement sensor cannot work normally in a liquid environment [10,11]. Therefore, these devices have been shown to have certain disadvantages in terms of the length of time of measurement, the large volume of the device, loss of gas, and a relatively higher error rate [12,13,14].

PZT piezoelectric transducers are high-sensitivity sensors for acoustic-electric measurement, mainly employed for photoacoustic measurement of liquid and solid [15,16,17] and allowing direct contact with the analyte [18,19,20,21]. They are commonly used in medical imaging [22], the study of nanoparticle suspensions [22], and photoacoustic imaging [23]. Apart from functioning as sensors for measuring thermal diffusivity [19,20,22], PZT piezoelectric transducers can also act as sensors for measuring acoustic vibration to obtain the concentration of trace gases [24]. In 2004, Ledermann carefully compared the structure of PZT thin-film cantilever beams with a bridge acoustic sensor and found that the measurement sensitivity of the former proved to be ppm [25] through an experiment involving the measurement of the concentration of CO_2_. In 2020, Kanchalar Keeratirawee et al. measured the concentration of NO_2_ using PZT piezoelectric transducers and the fitting rate reached 0.998 [26]. In sum, the PZT piezoelectric ceramic microphone, which acts as a PZT piezoelectric transducer, enables the collection of the photoacoustic signal generated by a gas in a liquid. Therefore, PZT piezoelectric-photoacoustic spectroscopy makes it possible to directly measure the gas concentration in a gas-liquid solution.

The novel contribution of the present study is that the current measuring device based on photoacoustic spectroscopy can only measure the concentration of a single phase (e.g., gas, liquid, solid); however, for the mixed phase state (gas-liquid), it needs to be equipped with a degassing device, which cannot directly measure the gas concentration. In this paper, the feasibility of the direct measurement of the concentration of CO_2_ in a gas-liquid solution, based on PZT piezoelectric-photoacoustic spectroscopy, was investigated experimentally in order to apply photoacoustic spectroscopy to the field of measurement of concentration in mixed phase (gas-liquid) in the future.

## 2. The Device and Method of the Experiment

**Figure 1:**
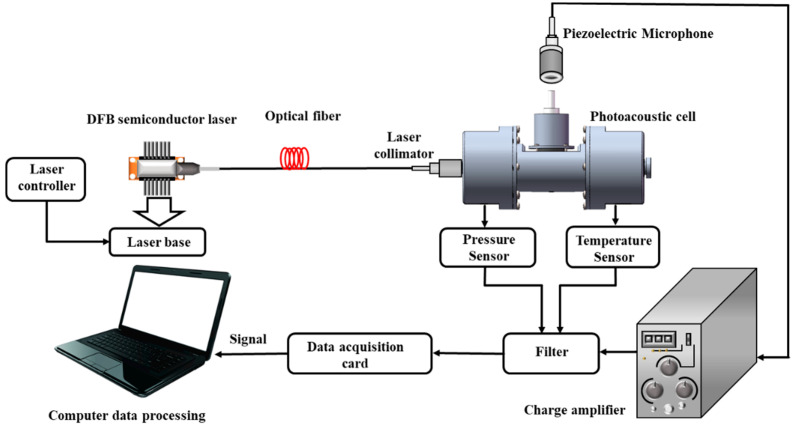
Structure diagram of the device.

The measuring device proposed in this paper is mainly composed of a laser signal modulation system, a photoacoustic signal acquisition system and a data processing system. Its structure diagram is shown in Figure 1. The function of the photoacoustic signal modulation system is to control the laser power, output wavelength and output pulse frequency. The photoacoustic signal acquisition system consists of a closed liquid photoacoustic cell and a PZT piezoelectric ceramic microphone. The collected photoacoustic signal is transmitted to the data acquisition card after being amplified by the charge amplifier, which is followed by data transmission from the acquisition card to the PC for storage, processing and display.

### 2.1. Method

**Figure 2:**
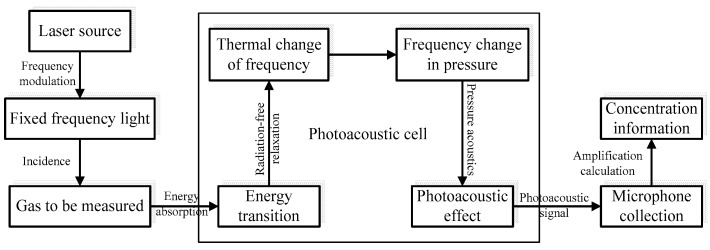
Schematic diagram of photoacoustic spectroscopy.

Unlike the traditional method of measurement, which mainly utilizes absorption spectroscopy, and the device usually measures the analyte’s power of absorbed light after the laser has passed through a certain path, measurement based on photoacoustic spectroscopy is different in terms of both theory and methodology. The schematic diagram is shown in Figure 2. The analyte first enters a closed photoacoustic cavity. Here, with a light source of radiation inducing it to absorb energy, the molecule of the analyte jumps from a low energy level to a high one. However, the high energy level of the analyte molecule eventually returns to a low energy level as its energy is distributed through its own release and non-radiation relaxation. Moreover, the molecular energy is converted into translational energy during the non-radiation relaxation process, which causes the temperature of the analyte to rise. In addition, the frequency of the temperature change of the analyte in the photoacoustic cell can be kept the same as the frequency of modulation by modulating the frequency of the incident light. The temperature change in the photoacoustic cell results in a pressure change in the photoacoustic cell, which causes the emergence of the acoustic signal. The acoustic sensor converts the acoustic signal into a photoacoustic signal. The concentration of the analyte can be accurately calculated using the known proportional relationship between the intensity of the photoacoustic signal and the concentration of the analyte.

Given that the laser intensity of the incident beam is I(r,t) and the frequency is ω, the heat source H(r,t) generated from the light energy absorbed by the sample gas can be expressed by the following formula under the condition of unsaturated absorption:

(1) H(r,t)=αI(r,t)

In Formula (1), α is the absorption coefficient of the sample

(2) α=Nδ

In Formula (2), δ is the absorption cross-section of the molecular. From Formula (2), it can be seen that, for the light wave whose wavelength is λ, its absorption coefficient α is a function of N (the absorption molecular concentration) and the absorption cross-section which describes the molecular transition characteristics. If δ is known, along with the wavelength of the incident light being λ, N can be determined by the strength of H(r,t).

### 2.2. Laser Signal Modulation System

**Figure 3:**
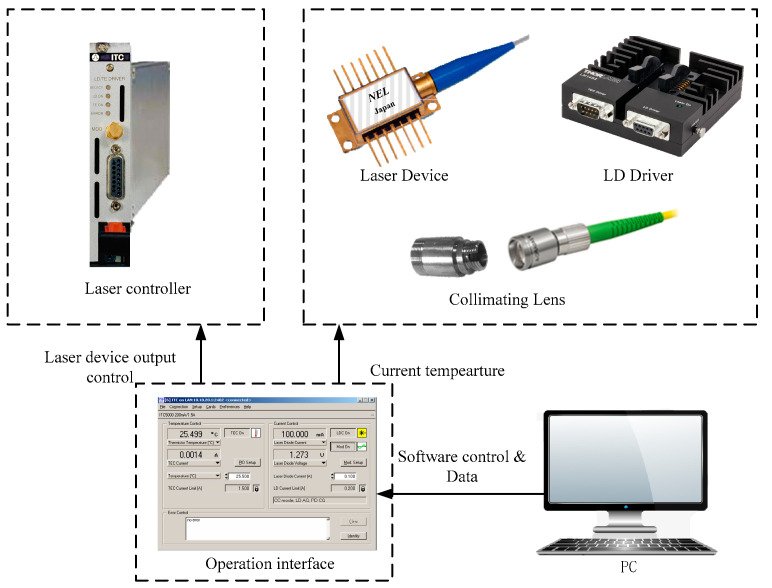
Structure diagram of the laser modulation system.

The laser signal modulation system includes a laser controller, a near-infrared fixed-wavelength DFB semiconductor laser (1580.35 nm), and an optical focusing element. Its structure diagram is shown in Figure 3. The DFB semiconductor laser installed on the heat dissipation base is controlled by a laser controller which can output a pulsed laser with a fixed power, wavelength and frequency by modulation of the temperature and current of the laser. The laser light enters the photoacoustic cell after being collimated by the collimator. Furthermore, since the purpose of the device is to directly measure the concentration of CO_2_ in the gas-liquid solution, the collimator is installed on the photoacoustic cell in order to prevent the laser from propagating through two kinds of media. In addition, disturbing noise caused by the deviation of the laser path can be avoided by the alignment of the collimator to the photoacoustic cavity because it can ensure that the laser light propagates in a straight line in the photoacoustic cell.

### 2.3. The Photoacoustic Signal Acquisition System

The photoacoustic signal acquisition system comprises two main parts: a liquid photoacoustic cell and a PZT piezoelectric ceramic microphone. The collection of the photoacoustic signal is accomplished by the PZT piezoelectric ceramic microphone and plays an essential role in the measurement of CO_2_ concentration. In addition, the size of the photoacoustic cell has a direct impact on the resonant frequency of the photoacoustic cell, the intensity of the photoacoustic signal generated by the analyte in the photoacoustic cell, and the sensitivity of the measuring device [3,27].

**Figure 4:**
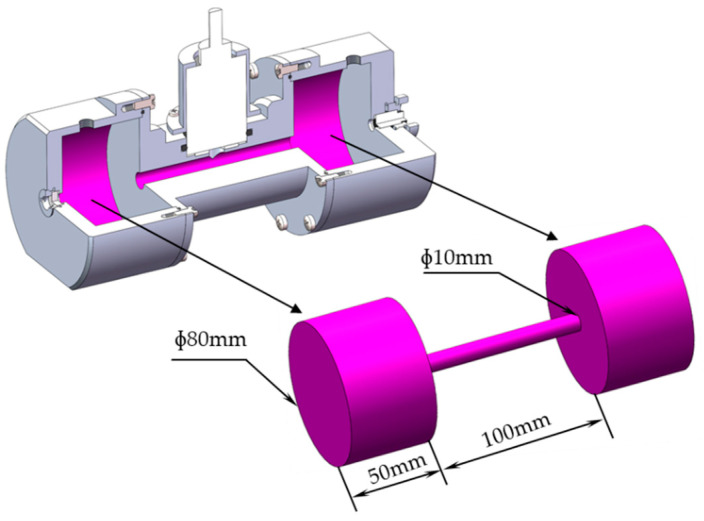
The structure of the photoacoustic cell.

The liquid photoacoustic cell adopts a cylindrical structure. Its structure is shown in Figure 4. The symmetrical structure of the cylindrical photoacoustic cell facilitates stable transmission of the laser. At the same time, a less demanding manufacturing process makes it ideal to use in the experiment.

When ω (the modulation frequency of the laser) is less than $\omega_{0}$ (the resonance frequency of the photoacoustic cell), the photoacoustic cell is in a non-resonant mode. Based on the condition above, it can be assumed that the pressure in the whole photoacoustic cell is almost the same. The sound pressure is evenly distributed in the photoacoustic cell [28,29,30]. The intensity of the photoacoustic signal in the photoacoustic cell can be expressed as:

(3) $$A\left(\omega \right)=\frac{\pi^{2}}{\sqrt{2}}SC\frac{\alpha \beta }{C_{p}}\cdot \frac{I_{0}\sum_{m=0}^{l}R\left(r,r_{0}\right)A_{m}\left(r,r_{0},\omega \right)}{\omega }$$

(4) $$l=∥\frac{r_{0}\omega }{2SC}∥$$

(5) $$R\left(r,r_{0}\right)=\frac{1}{r_{0}}e^{-\frac{2s^{2}r^{2}}{r_{0}^{2}}}[I_{0}^{2}\left(\frac{2s^{2}r^{2}}{r_{0}^{2}}\right)+\frac{1}{\pi^{2}}K_{0}^{2}\left(\frac{2s^{2}r^{2}}{r_{0}^{2}}\right){]}^{1/2}$$

(6) $$A_{m}\left(r,r_{0},\omega \right)=e^{-\frac{4S^{2}C^{2}m^{2}}{r_{0}^{2}\omega^{2}}}I_{0}\left(\frac{8S^{2}rCm}{r_{0}^{2}\omega^{2}}\right)$$

**Figure 5:**
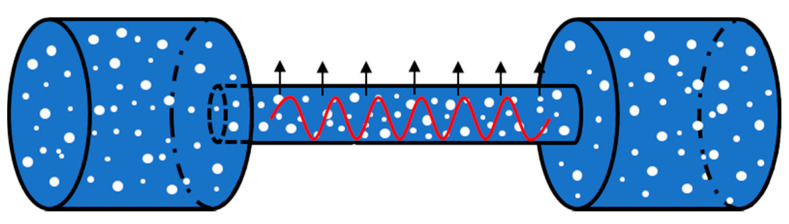
Photoacoustic signal in liquid photoacoustic cell.

In Formula (3), S is the light scattering factor, C is the speed of sound in the bubble-containing liquid, β is the isothermal expansion coefficient, and $C_{p}$ is the specific heat at constant pressure, *m* is the order of higher harmonics, *r* is the radius of the photoacoustic cell, $r_{0}$ is the radius of the laser beam. The photoacoustic signal in the liquid photoacoustic cell is shown in Figure 5.

When the modulation frequency of the laser is not greater than 1 kHz, then l=0, and the intensity of the photoacoustic signal can be expressed as:

(7) $$A\left(\omega \right)=\xi \cdot \frac{I_{0}R\left(r,r_{0}\right)}{\omega }$$

(8) $$\xi =\frac{\pi }{\sqrt{2}}SC\frac{N\delta \beta }{C_{p}}$$

In Formula (7), $I_{0}$ is the maximum intensity of the laser, ξ is the coefficient which represents the conversion of light energy into mechanical energy, through which the concentration of the gas can be obtained.

**Figure 6:**
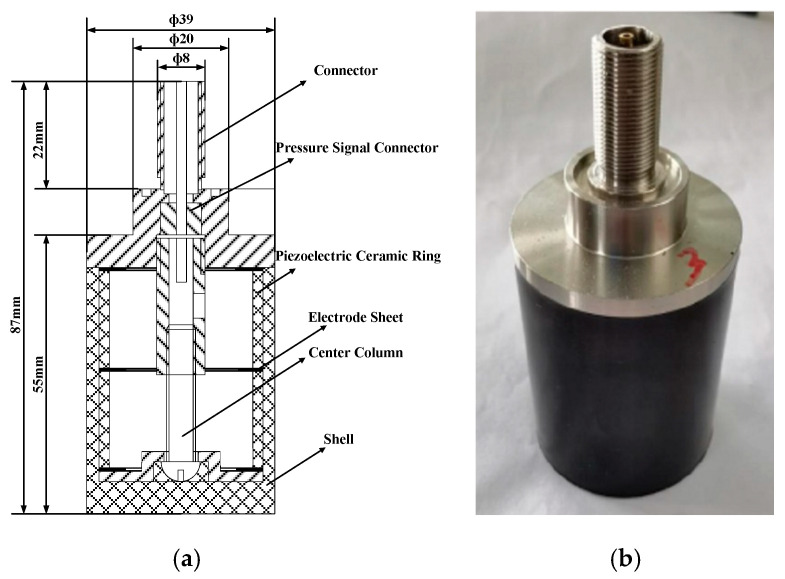
(**a**) Plane structure of piezoelectric ceramic microphone; (**b**) Physical picture of piezoelectric ceramic microphone.

The working principle of a piezoelectric transducer is the piezoelectric effect. The piezoelectric effect is observed when deformation of the piezoelectric material causes the electric polarization of the material to change. That is, when the piezoelectric material is subjected to a mechanical load, the charges appear on some opposite surfaces of the piezoelectric material [31]. The sensitivity of the PZT piezoelectric ceramic microphone designed in this article is (−150 ± 2) dB, and the resonance frequency is (27 ± 2) kHz. Structural and physical pictures are shown in Figure 6. Compared with traditional condenser microphones, PZT piezoelectric ceramic microphones can utilize all the light energy absorbed by the sample in a more effective way. Therefore, it is easier to measure samples with general absorption characteristics compared with gas microphones.

### 2.4. Data Processing System

**Figure 7:**
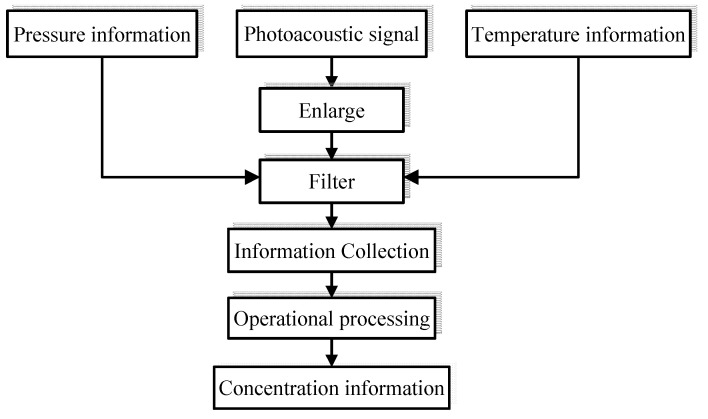
Flowchart of data processing system.

The data processing system of this device is mainly composed of a charge amplifier, R/C filter, data acquisition card and computer. The charges collected by the PZT piezoelectric ceramic microphone are amplified by the charge amplifiers, which is followed by the filtering of the output voltage signal through a filter and then input to the data acquisition card. In addition to collecting signals from the PZT piezoelectric ceramic microphone, the data acquisition card also collects data from pressure sensors and temperature sensors. What the data acquisition card collects is input into the computer for arithmetic processing. Finally, the gas concentration is calculated. The flowchart of the data processing system is shown in Figure 7.

## 3. Experiment

The first experiment sought to analyze the relationship between the intensity of a photoacoustic signal and the concentration of CO_2_. The analyte to be measured by the device proposed in this article was the standard gas-liquid solution with a concentration of CO_2_ ranging from 3 mL/L to 21 mL/L. The output frequency of the pulsed laser was 20 Hz. The experimental temperature was kept at 27 °C, and the current at 70 mA. The temperature of the solution was maintained at room temperature, and the pressure in the photoacoustic cell was standard atmospheric pressure. Each measurement required the following steps:

1. Preheating of the charge amplifier for 30 min, and exporting of the charge of the PZT piezoelectric ceramic microphone before connection to the charge amplifier;
2. Cleaning of the inner wall of the photoacoustic cell, then letting in inert gas for ten minutes. Setting the temperature of the laser at room temperature, with an output current of 70 mA. Collection occurred until the laser output power was stabilized;
3. Pouring a standard CO_2_ solution into the photoacoustic tank through a water pump. During this period, controlling the pressure sensor and temperature sensor in order to ensure that the pressure in the tank was standard atmospheric pressure and the temperature was room temperature after the solution was flushed each time.

How variation in the laser modulation frequency influenced the intensity of the photoacoustic signal was also studied in this experiment using the device proposed in this paper. The modulation frequency of the laser varied from 20 Hz to 1 kHz. With the concentration of CO_2_ in the solution being 6.6 mL/L, the measurements were carried out according to the following procedures:

1. Filling of the photoacoustic cell with high-purity N_2_ for ten minutes in case gaseous impurities remained;
2. Pouring the solution, in which the concentration of CO_2_ was 6.6 mL/L, into the closed photoacoustic cell at room temperature;
3. Irradiating the pulsed laser, with frequency 20 Hz–1 kHz, into the photoacoustic cell, and observing the response of the photoacoustic signal under different modulation frequencies.

## 4. Experimental Results

**Figure 8:**
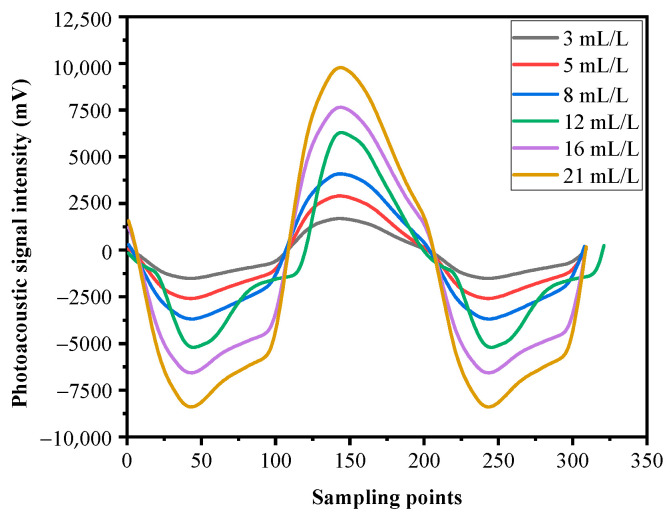
Photoacoustic signals at different concentrations.

**Figure 9:**
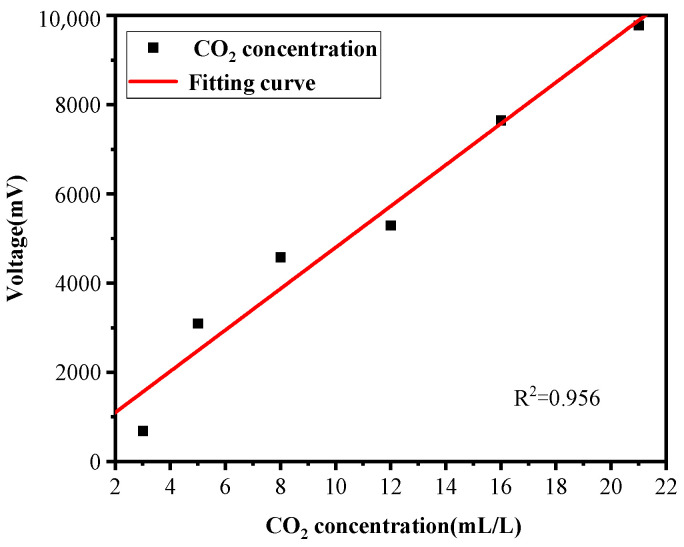
Calibration curves.

The first experiment mainly sought to analyze how the intensity of photoacoustic signal changes as the concentration of CO_2_ varies. The data acquisition card collected 100 sets of data, each of which included 10,240 sampling points. Each sampling point corresponded to the voltage values output by the charge amplifier. The peak voltage value of each set of data was selected and then the average value calculated, being the voltage for different concentrations of CO_2_. The results are shown in Figure 8. Figure 9 shows the calibration curves of different CO_2_ concentrations versus photoacoustic signal intensity.

**Figure 10:**
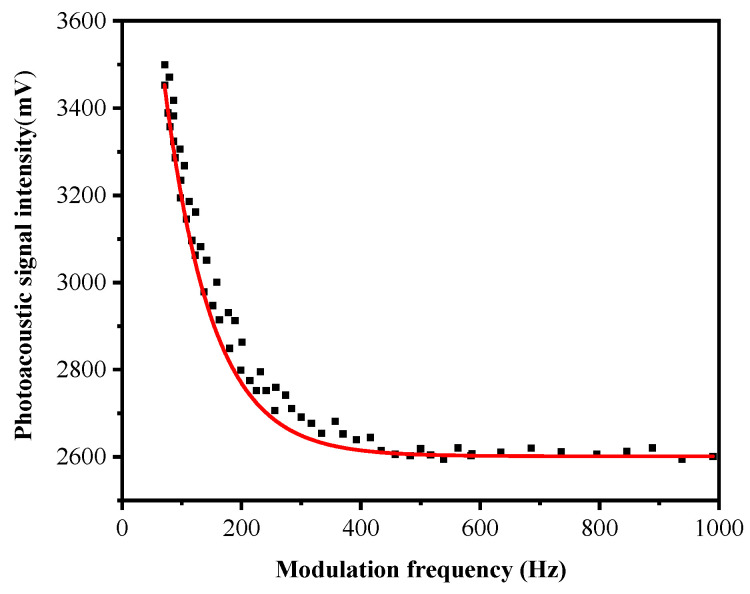
Photoacoustic signals under different modulation frequencies.

The second experiment was conducted for the purpose of analyzing how the variation of the laser modulation frequency affects the intensity of photoacoustic signal. We collected 100 sets of data, each of which included 10,240 sampling points obtained by the charge amplifier at each frequency. The peak voltage of each set of data was selected and then the average value calculated. Finally, the curve of the photoacoustic signal intensity under different modulation frequencies was drawn. Figure 10 shows the response of the photoacoustic signal under different modulation frequencies.

**Figure 11:**
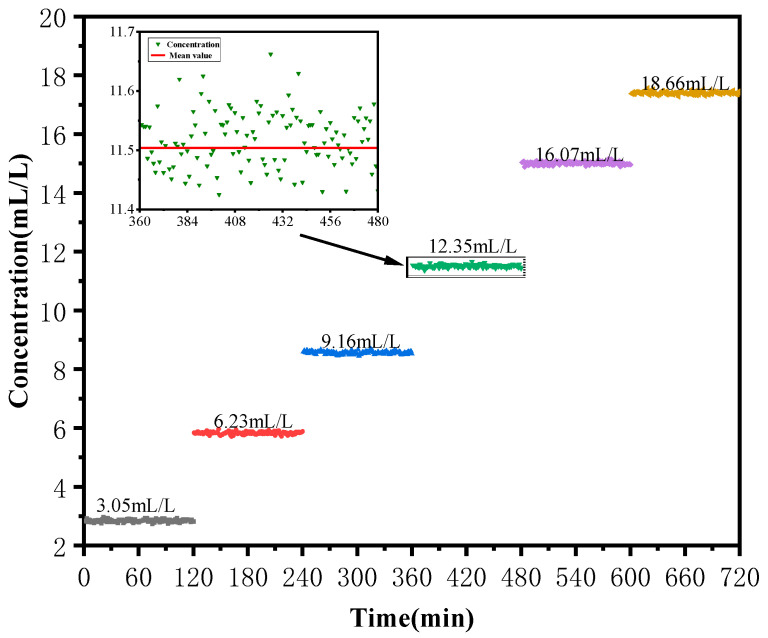
Experimental results for the measurements.

The third experiment was carried out to measure the concentration of CO_2_ in standard gas-liquid solution using the device proposed in this paper. The concentrations of CO_2_ in the standard solution were 3.05 mL/L, 6.23 mL/L, 9.16 mL/L, 12.35 mL/L, 16.07 mL/L and 18.66 mL/L. The output frequency of the pulsed laser was 20 Hz, the temperature was 27 °C, and the current was 70 mA. Each of the CO_2_ solutions referred to above were measured 120 times, one by one, and on each occasion the measurement was for less than one minute. The solution was kept at room temperature, and the pressure in the photoacoustic cell was standard atmospheric pressure. The measurement results are shown in Figure 11 and the specific data is displayed in Table 1.

## 5. Discussion and Conclusions

From Figure 8, the conclusion can be drawn that the intensity of the photoacoustic signal gradually increased as the concentration of CO_2_ in the solution increased, which is consistent with results described in [5]. The photoacoustic signal collected by the PZT piezoelectric ceramic microphone changed with the concentration of CO_2_; specifically, the intensity of the photoacoustic signal was found to be proportional to the concentration of CO_2_. Therefore, the measuring device proposed in this paper was able to directly measure the concentration of CO_2_ in gas-liquid solution.

From Figure 10, it can be inferred that the intensity of the photoacoustic signal gradually decreased as the laser modulation frequency increased. When the laser modulation frequency was above 100 Hz, the intensity of the CO_2_ photoacoustic signal hardly changed. This does not conform to the inverse relationship between the intensity of the photo-acoustic signal and the modulation frequency of the laser described in Equation (6) in the text. The reason is that when the laser modulation frequency is above 400 Hz, the response of the PZT piezoelectric ceramic microphone to the intensity change of the photoacoustic signal of CO_2_ is not obvious. Since previous measurement devices include a degassing component, this generates a different experimental result [32]. This can be primarily explained by the fact that CO_2_ is in a different environment. Secondly, the photoacoustic cell used in [32] operates in a resonance mode (1.7 kHz), while the device proposed in this paper operates in a non-resonant mode (20 Hz). Moreover, the results regarding the relationship between the modulation frequency of the laser and the photoacoustic signal intensity obtained from the experimental research on the measurement of gas concentration in pure liquid based on photoacoustic spectroscopy is similar to what has been recorded in the literature [33].

It is evident from Table 1 that the overall measurement error of the device was under 7%, which is obtained from comparison of the average value of the concentration of CO_2_ obtained from experimental measurement and the standard value of the concentration of CO_2_ represented by the original standard solution. Therefore, the device proposed in this paper can meet the requirement of measurement accuracy. On the other hand, as is shown in Table 1, it is clear that the average value of the concentration of CO_2_ in each solution was lower than that of the standard solution, which can be explained by the loss of CO_2_ caused by the liquid pump at the very beginning of the experiment. Finally, figures in Table 1 demonstrate that the time of each measurement was less than 60 s, which saved at least 10 min compared with devices proposed in the literature [34,35].

In conclusion, the series of experiments undertaken confirm that the scheme proposed in this paper is feasible. Above all, it has been demonstrated that the concentration of CO_2_ in the gas-liquid solution is proportional to the intensity of the photoacoustic signal generated by the CO_2_. Furthermore, the photoacoustic signal of CO_2_ gradually declines as the laser modulation frequency increases. In addition, compared with the traditional experimental device, the device proposed in this article can shorten the measurement time to tens of seconds due to the removal of the degassing component. In the future, research will focus on improvement of the device in terms of sensitivity and its lower limit of measurement. By experiment, this paper has only studied the measurement of the concentration of a single-component gas (CO_2_) in gas-liquid solution. For future study, the focus may include application of the device to the measurement of multi-component gas concentration in gas-liquid solution.

## Figures and Tables

**Table 1:** Specific measurement data.

Standard Value (mL/L)	Average Value (mL/L)	Relative Error (%)	Time of a Single Measurement (s)
3.05	2.85	6.4	54
6.23	5.82	6.5	56
9.16	8.56	6.5	52
12.35	11.51	6.7	53
16.07	15.01	6.6	55
18.66	17.39	6.8	54

## Data Availability

The data presented in this study are available on request from the corresponding author.

## References

[1] Peng Z., Lee-Taylor J., Orlando J.J., Tyndall G.S., Jimenez J.L. (2019). Organic peroxy radical chemistry in oxidation flow reactors and environmental chambers and their atmospheric relevance. Atmos. Chem. Phys. Discuss..

[2] Hu Y., Qiao S., He Y., Lang Z., Ma Y. (2021). Quartz-enhanced photoacoustic-photothermal spectroscopy for trace gas sensing. Opt. Express.

[3] Yuan C., Ruifeng W., Jie P., Liu K., Chen W., Wang G., Gao X. (2021). Humidity enhanced N_2_O photoacoustic sensor with a 4.53 μm quantum cascade laser and Kalman filter. Photoacoustics.

[4] Dumitras D.C., Petrus M., Bratu A.-M., Popa C. (2020). Applications of Near Infrared Photoacoustic Spectroscopy for Analysis of Human Respiration: A Review. Molecules.

[5] Qian S., Tian C., Wang Z., Yu Y., Zhang X. (2021). Elimination of laser power loss influence for multi-point gas sensing in photoacoustic spectroscopy. IEEE Sens. J..

[6] Wu Z., Zhang Y., Zhang H., Gai N. (2019). A photoacoustic spectroscopy measurement of near-infrared low-mass multi-component gas at room temperature and atmospheric pressure. Measurement.

[7] Gong Z., Wu G., Jiang X., Li H., Gao T., Guo M., Ma F., Chen K., Mei L., Peng W. (2021). All-optical high-sensitivity resonant photoacoustic sensor for remote CH_4_ gas detection. Opt. Express.

[8] Thaler K.M., Niessner R., Haisch C. (2017). Laboratory and field studies on a new sensor for dissolved N_2_O. Anal. Bioanal. Chem..

[9] Liu L., Huan H. (2020). Multiple Dissolved Gas Analysis in Transformer Oil Based on Fourier Transform Infrared Photoacoustic Spectroscopy. Spectrosc. Spectr. Anal..

[10] Ke C., Min G., Beilei Y., Feng J., Wang G., Ma F., Li C., Zhang B., Deng H., Gong Z. (2021). Highly Sensitive Optical Fiber Photoacoustic Sensor for In Situ Detection of Dissolved Gas in Oil. IEEE Trans. Instrum. Meas..

[11] Zakrzewski J., Maliński M., Chrobak Ł., Pawlak M. (2016). Comparison of Theoretical Basics of Microphone and Piezoelectric Photothermal Spectroscopy of Semiconductors. Int. J. Thermophys..

[12] Jiang J., Wang Z., Han X., Zhang C., Ma G., Li C., Luo Y. (2019). Multi-gas detection in power transformer oil based on tunable diode laser absorption spectrum. IEEE Trans. Dielectr. Electr. Insul..

[13] Mao X., Zhou X., Zhai L., Yu Q. (2015). Dissolved Gas-in-Oil Analysis in Transformers Based on Near-Infrared Photoacoustic Spectroscopy. Int. J. Thermophys..

[14] Sepulveda-Jauregui A., Martinez-Cruz K., Strohm A., Anthony K.M.W., Thalasso F. (2012). A new method for field measurement of dissolved methane in water using infrared tunable diode laser absorption spectroscopy. Limnol. Oceanogr. Methods.

[15] Laufer J., Jathoul A., Pule M., Beard P. (2013). In vitro characterization of genetically expressed absorbing proteins using photoacoustic spectroscopy. Biomed. Opt. Express.

[16] He Q., Zhang Q., Cao W., Yin T., Zhao S., Yin X., Zhao H., Tao W. (2019). Detecting trace of mercury ions in water using photoacoustic method enhanced by gold nanospheres—Sciencedirect. Microchem. J..

[17] Sun Q.-M., Gao C.-M., Zhao B.-X., Rao H.-B. (2010). Thermal diffusivity of light-emitting diode packaging material determined by photoacoustic piezoelectric technique. Chin. Phys. B.

[18] Hernandez-Aguilar C., Dominguez-Pacheco A., Valderrama-Bravo C., Cruz-Orea A., Ortiz E.M., Ordonez-Miranda J. (2020). Photoacoustic Spectroscopy in the Characterization of Bread with Turmeric Addition. Food Bioprocess Technol..

[19] Bodzenta J., Pustelna B., Kleszczewski Z. (1993). Photoacoustic imaging of ion-implanted semiconductor samples. Ultrasonics.

[20] Chen J., Zhang L., Liang Y.-C. (2019). Exploiting Gaussian Mixture Model Clustering for Full-Duplex Transceiver Design. IEEE Trans. Commun..

[21] Bell K., Reza P.H. (2020). Non-contact reflection-mode optical absorption spectroscopy using photoacoustic remote sensing. Opt. Lett..

[22] Dubyk K., Pastushenko A., Nychyporuk T., Burbelo R., Isaiev M., Lysenko V. (2018). Thermal conductivity of silicon nanomaterials measured using the photoacoustic technique in a piezoelectric configuration. J. Phys. Chem. Solids.

[23] Kitai T., Torii M., Sugie T., Kanao S., Mikami Y., Shiina T., Toi M. (2012). Photoacoustic mammography: Initial clinical results. Breast Cancer.

[24] Lv H., Luo H., Zheng H., Zhu W., Fang J., Yu J., Tittel F., Chen Z. (2021). Application of piezoelectric transducers in photoacoustic spectroscopy for trace gas analysis. Microw. Opt. Technol. Lett..

[25] Ledermann N., Muralt P., Baborowski J., Forster M., Pellaux J.P. (2004). Piezoelectric pb(zrx, ti1-x)o3 thin film cantilever and bridge acoustic sensors for miniaturized photoacoustic gas detectors. J. Micromech. Microeng..

[26] Keeratirawee K., Hauser P.C. (2020). Piezoelectric tube as resonant transducer for gas-phase photoacoustics. Anal. Chim. Acta.

[27] Cao Y., Liu Q., Wang R., Liu K., Chen W., Wang G., Gao X. (2021). Development of a 443 nm diode laser-based differential photoacoustic spectrometer for simultaneous measurements of aerosol absorption and NO_2_. Photoacoustics.

[28] Luo J., Fang Y.H., Zhao Y.D., Wang A.J., Li D.C., Li Y.Y., Liu Y., Cui F.X., Wu J., Liu J.X. (2014). Research on the detection of SF6 decomposition products based on non-resonant photoacoustic spectroscopy. Anal. Methods.

[29] Sawada T. (1988). Principles and Applications of Photoacoustic Spectroscopy.

[30] Yin Q. (1991). Photoacoustic Photothermal Technology and Its Application.

[31] Russo S.D., Zhou S., Zifarelli A., Patimisco P., Sampaolo A., Giglio M., Iannuzzi D., Spagnolo V. (2020). Photoacoustic spectroscopy for gas sensing: A comparison between piezoelectric and interferometric readout in custom quartz tuning forks. Photoacoustics.

[32] Yin X., Gao M., Miao R., Zhang L., Zhang X., Liu L., Shao X., Tittel F.K. (2021). Near-infrared laser photoacoustic gas sensor for simultaneous detection of CO and H_2_S. Opt. Express.

[33] Marín E., Vera-Medina G., Garcia A., Calderon A. (2010). On the modulation frequency dependence of the photoacoustic signal for a metal coated glass-liquid system. Open Phys..

[34] Chen T., Ma F., Zhao Y., Zhao Y., Wan L., Li K., Zhang G. (2021). Portable ppb-level acetylene photoacoustic sensor for transformer on-field measurement. Optik.

[35] Chen Y., Wang Z., Li Z., Zheng H., Dai J. (2021). Development of an Online Detection Setup for Dissolved Gas in Transformer Insulating Oil. Appl. Sci..

